# HSPA8/HSC70 in Immune Disorders: A Molecular Rheostat that Adjusts Chaperone-Mediated Autophagy Substrates

**DOI:** 10.3390/cells8080849

**Published:** 2019-08-07

**Authors:** Srinivasa Reddy Bonam, Marc Ruff, Sylviane Muller

**Affiliations:** 1 Neuroimmunology & peptide therapy, Biotechnology and cell signaling, CNRS-University of Strasbourg, Illkirch 67412, France/Laboratory of excellence Medalis, 67000 Strasbourg, France; 2 Biologie Structurale Intégrative, Institut de Génétique et de Biologie Moléculaire et Cellulaire, Illkirch, 67404 Strasbourg, France; 3 University of Strasbourg Institute for Advanced Study (USIAS), 67000 Strasbourg, France; 4 Fédération Hospitalo-Universitaire (FHU) OMICARE, Fédération de Médecine Translationnelle de Strasbourg (FMTS), Strasbourg University, 67000 Strasbourg, France

**Keywords:** chaperone-mediated autophagy, HSPA8/HSC70, lysosomes, pharmacological regulators, P140, autoimmune diseases, systemic lupus erythematosus

## Abstract

HSPA8/HSC70 is a molecular chaperone involved in a wide variety of cellular processes. It plays a crucial role in protein quality control, ensuring the correct folding and re-folding of selected proteins, and controlling the elimination of abnormally-folded conformers and of proteins daily produced in excess in our cells. HSPA8 is a crucial molecular regulator of chaperone-mediated autophagy, as a detector of substrates that will be processed by this specialized autophagy pathway. In this review, we shortly summarize its structure and overall functions, dissect its implication in immune disorders, and list the known pharmacological tools that modulate its functions. We also exemplify the interest of targeting HSPA8 to regulate pathological immune dysfunctions.

## 1. Introduction

Heat shock proteins (HSP) constitute a large family of highly homologous chaperone proteins that are induced in response to an elevation of temperature (hence their original name), but more generally to environmental, physical and chemical stresses as diverse as cold, ultraviolet irradiation and wound healing. Their rapid expression limits the consequences of any damages induced by these stresses and facilitates cell recovery. The HSP70 family of chaperones, one of the most ubiquitous classes of chaperones, is composed of at least 13 members, including stress-induced proteins such as HSPA1A [HSP70-1/HSP72; 72 kDa; 641 amino acid (aa) residues], and members that are not stress-inducible, such as HSPA5 (GRP78/Bip/Mif-2; 78 kDa; 654 aa), HSPA8 (HCC70/HSP73/HSP71; 73 kDa; 646 aa) and HSPA9 (mtHSP70/HSP75/GRP75/mortalin/; 75 kDa; 679 aa). Among these HSPs, HSPA1A and HSPA8 are located in the cytosol, the nucleus, extracellular exosomes, and at the cell membrane, HSPA5 is present in the endoplasmic reticulum and extracellular exosomes, while HSPA9 is found in the mitochondria and in the nucleus [1]. More detailed information about the HSPs’ sequence homology and identity, their location and main functions can be found in our previous [2] and other comprehensive reviews [1,3]. 

Major similarities and differences between the stress-inducible HSPA1A and the constitutively-expressed HSPA8 proteins, which display 85% identity in their human form, are summarized in the Table 1.

**Figure 1:**
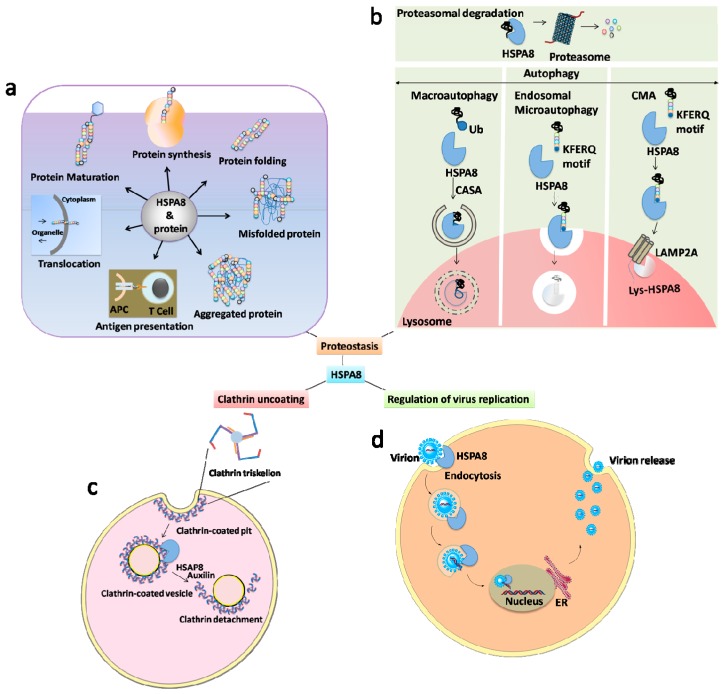
The broad spectrum of HSPA8 functions. HSPA8 with its co-chaperones and cooperating chaperones constitute a complex network of folding machines. It is therefore involved in many decisive aspects of cell survival. (**a**) Protein regulation: HSPA8 is a well-known protein regulator in terms of folding, maturation, translocation, assembly, disassembly, aggregation, antigen presentation and differentiation. (**b**) HSPA8 is determining in autophagy (CASP, CMA, microautophagy) and ubiquitin–proteasome system. (**c**) Uncoating of clathrin-coated pits: HSPA8 uncoates the clathrin triskelions from clathrin-coated pits, which are involved in several processes of intracellular pathways (e.g., cycles of endocytosis of the synaptic vesicles and receipts trafficking). This proceeds in an ATP-dependent manner with the help of auxilin. (**d**) HSPA8 is also known to be involved in the regulation on virus replication (both positive and negative). Abbreviations: APCs, antigen-presenting cells; CMA, chaperone-mediated autophagy; CASA, chaperone-assisted selective autophagy; ER, endoplasmic reticulum; LAMP2A, lysosomal-associated membrane protein 2A; Lys-, lysosomal; Ub, ubiquitin.

In the large HSP70 family, the HSPA8 chaperone plays a major role in the protein quality control system (reviewed in [1,2,3,4,5]). It acts as a folding catalyst of proteins assuring the re-folding of misfolded conformers, and as a controller targeting proteins for subsequent degradation. These functions all appear to be based upon the property of HSPA8 to interact with hydrophobic segments in an ATP-controlled manner. HSPA8 is notably involved in the presentation of antigenic peptides by major histocompatibility complex (MHC) class II (MHCII) molecules to CD4^+^ T cells [6,7]. It also acts as an adenosine 5’-triphosphatase (ATPase) in the disassembly of clathrin-coated vesicles during the transport of membrane components in the cells [8,9,10,11]. It plays a pivotal role in autophagy, most particularly—but not only—in the process of chaperone-mediated autophagy (CMA) (Figure 1).

Recent findings have emphasized the involvement of HSPA8 in several vital functions of the cells, notably as a regulator of lysosome activity, and therefore as a modulator of autophagy processes. Dysfunction of lysosomes and autophagy activity in diseases are also better known. Based upon these recent progresses, promising novel opportunities for therapeutic intervention through HSPA8 are emerging. After briefly over viewing the HSPA8 structure and functions, this short article describes the role of HSPA8 in immune disorders, and presents the current ‘toolbox’ of pharmacological agents that might be effective therapeutics in this set of indications. It might also help to contribute at the development of new pharmacological tools in the context of cancer, neurological/neurodegenerative diseases, metabolic diseases and aging, which are outside the scope of this article, but in which HSPA8 also plays critical roles (reviewed in [3,12,13,14]).

## 2. HSPA8 and Autophagy

CMA, in which HSPA8 is a key component, is a cellular lysosome-mediated degradative mechanism that displays particular characteristics. One of these is that substrates reach the lumen of lysosomes directly by a pathway that does not require intermediate vesicles (Figure 1) [15].

In the canonical macroautophagy (Figure 1) process, a double-membrane sequestering compartment termed “phagophore” is formed, which matures into a double-membrane vacuole termed “autophagosome” that contains cytoplasmic substrates subjected to further processing. 

The mechanisms of autophagosome formation have been shown recently to implicate coat protein complex II (COPII) vesicles as a membrane source [16,17]. Autophagosomes subsequently fuse with hydrolytic enzyme-rich lysosomes to form vacuoles called “autolysosomes”, in which the engulfed cellular cargos are cleaved. The resulting compounds are released back into the cytosol for reuse (recycling) [14]. Autophagosomes can also fuse with single-membrane endosomes to give amphisomes (a single-membrane compartment), that converge with lysosomes to generate autolysosomes [18]. Besides macroautophagy, by far the most extensively-studied form of autophagy, the delivery of autophagic cargo to lysosomes can also occur via microautophagy, known as endosomal microautophagy (eMI) in mammals (Figure 1). The latter is a process in which the lysosomal membrane invaginates to internalize cytosolic substrates into small vesicles, which then detach into the lysosomal lumen for degradation [19]. With the advancement of knowledge, it turns out that macroautophagy and microautophagy contribute to the degradation of organelles and proteins in a more selective manner than initially thought. Recent data have demonstrated in particular that epigenetic phenomena play a great role in regulating autophagy [20,21,22,23]. Histones encompassing post-translational modifications (notably in the N-terminus of histone H4) and non-coding microRNAs importantly contribute to the transcriptional and post-transcriptional control of autophagic flux. Post-translational modifications of autophagy-related proteins and also of substrates have an important role in the selectivity of these processes.

CMA does not involve vesicles, but instead implicates chaperone proteins to directly target specific proteins to the lumen of lysosomes ([24], reviewed in [14]). CMA targets proteins that possess a so-called “CMA-targeting recognition motif” of sequence KFERQ. This motif, which is present in ~40% of proteins in the mammalian proteome, is recognized by HSPA8, with a co-chaperone complex containing HSP90, HSP40, HSP70-interacting protein (HIP) and HSP70-HSP90 organizing protein (HOP). This heterologous chaperone/co-chaperone complex participates to substrate unfolding and delivers unfolded substrates to a protein named as the lysosome-associated membrane protein 2A (LAMP2A) receptor at the lysosomal membrane (Figure 1). LAMP2A forms a protein translocation complex, which binds substrates via its cytosolic C-terminal sequence, a 12 amino-acid-residues-long sequence, an interaction that does not involve the KFERQ-like motif. After its multimerization, LAMP2A internalizes the targeted proteins for the subsequent degradation into the lysosomal lumen. The acidic hydrolases present in the lysosomal lumen lyse proteins that are degraded for recycling, or that are transferred to the late endosomal MHC class II compartment (MIIC) to be loaded onto MHCII molecules and subsequently presented in this context to CD4^+^ T cells [25,26,27,28]. Thus, in CMA, two proteins display critical roles, and are considered as limiting effectors. They are HSPA8, which ensures the selectivity of proteins that will be degraded via the CMA pathway (cytosolic HSPA8), and which also contributes to the translocation of targeted substrates (lysosomal HSPA8/lys-HSPA8), and also LAMP2A, an integral component of the translocation complex, which is pivotal in the translocation of “tagged” cytosolic proteins across the lysosomal membrane (reviewed in [14]). HSPA8 and the CMA-targeted substrate bind the cytosolic LAMP2A tail simultaneously, suggesting that the substrate recognition and targeting processes are linked. Although this complex mechanism is now better identified and assessed, a deeper structure-function analysis of the interrelationships between HSPA8, LAMP2A and CMA-targeted substrates structure and functionality is necessary to gain a fine-tuned understanding of the mechanisms leading to substrate binding, LAMP2A assembly and substrate translocation [3,14].

Besides macroautophagy, microautophagy and CMA, there are several types of selective autophagy pathways that are involved in the degradation of various organelles, including mitochondria, peroxisomes, endoplasmic reticulum (ER), nucleus, and lysosomes [29,30,31]. Selective degradation of protein aggregates by aggrephagy, lipid droplets by lipophagy, glycogen by glycophagy or iron-sequestering protein ferritin by ferritinophagy, taken as examples, have been described [31,32,33]. Recent investigations have highlighted that HSPA8, a determining element of CMA, is also involved in these autophagy pathways, including macroautophagy and microautophagy/eMI [34]. 

In eMI, it has been shown for example that HSPA8 binds directly to lipids at the endosomal membrane without requiring LAMP2A for lysosomal docking [34,35]. In another form of autophagy called chaperone-assisted selective autophagy (CASA) (Figure 1) [36]), HSPA8 associated to Bcl-2-associated athanogene (BAG)1 and BAG3 co-chaperones participates to the degradation of ubiquitin-positive protein aggregates [12,37].

## 3. HSPA8 Structure and Structure-Function Relationships

**Figure 2:**
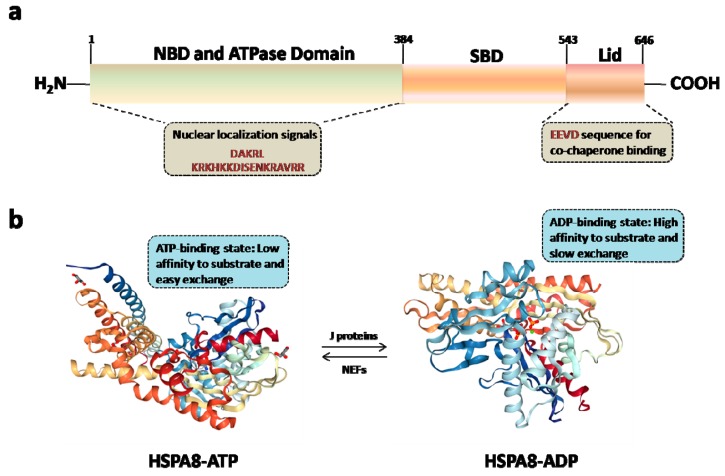

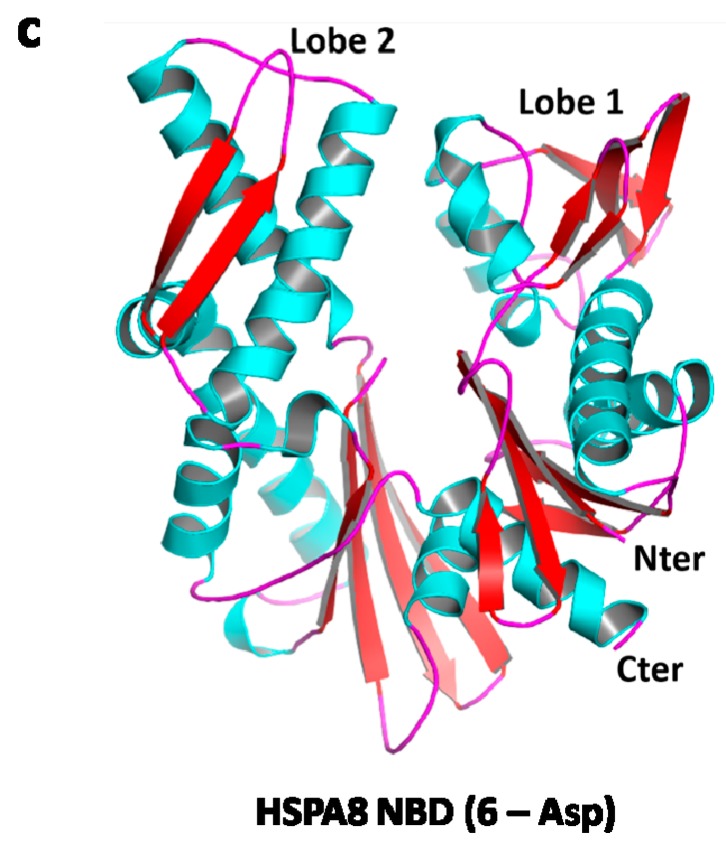
HSPA8 structure. (**a**) HSPA8 encompasses three main structural domains, namely the nucleotide-binding domain (NBD), which binds and hydrolyzes ATP, as well as a short hinge domain assuring flexibility between the two main domains, and thirdly, the substrate-binding domain (SBD) that contains two subdomains, namely a 15-kDa β-sandwich that binds peptide substrates, and a 10-kDa R-helix acting as a lid over the substrate binding site, and which is therefore central for chaperone/co-chaperone binding. Eukaryotic cytosolic HSPA8 contain a G/P-rich C-terminal region harboring an EEVD-motif involved in the binding of co-chaperones and other HSPs. (**b**) Structure of HSPA8 under two distinct states, namely ATP [PDB code 5AQM) and ADP [PDB code 4H5T) states with nucleotide exchange factors (NEFs) and J proteins, respectively [44,45]. (**c**) Overall structure of HSPA8 NBD (6—Asp). The NBD is divided in two lobes. The α-helices are depicted in blue, the β-sheets in red and the loops in purple (Ruff M. and Muller S., unpublished).

HSPA8 possesses a C-terminal protein substrate-binding domain (SBD) and a conserved N-terminal ATP-binding domain also called a nucleotide-binding domain (NBD) (Figure 2A; reviewed in [2]). At least two nuclear localizing signal sequences have been identified so far in human HSPA8, both located within the NBD. They are present in residues ^69^DAKRL^73^ in the N-terminus and ^246^KRKHKKDISENKRAVRR^262^ in the ATPase domain. A nucleolar localization signal, sufficient to promote nucleolar targeting in response to heat shock, was identified in residues 225–244 of HSPA8. Within the tertiary structure of NBD, this segment is located within the domain B of lobe II, which encompasses residues 229–306. A nuclear export signal motif has been identified in residues 394–401 of HSPA8 at the very N-terminus of the substrate-binding domain (reviewed in [38]).

Several structures of HSPA8 have been described so far [2,5]. HSPA8 that presents a modular structure with an hydrophobic hinge between the NBD and SBD, displays a large variability of conformation depending on the saline/pH environment, its state as monomers or oligomers, and on its association to many different ligands, substrates and cofactors [2,39]. In most of the partial crystal structures of the protein that have been generated, the lid domain in the C-terminal region (Figure 2A) moves over the substrate binding cavity, leading to its closure. Since this conformation does not allow substrate binding, in silico molecular modeling and docking studies with potential ligands have often been performed to model HSPA8 in open conformation where the lid is away from the substrate binding domain [40]. A structure of HSPA8, in the presence of ATP or ADP, is illustrated in Figure 2B [41,42]. Our own data are presented in Figure 2C (Ruff M. and Muller S., unpublished). Within the conserved NBD domain, several pockets have been identified that might be targeted for a possible inhibition of certain HSPA8 functionalities [41,43].

HSPA8 binds to small hydrophobic stretches of either nascent or partially-unfolded proteins in an ADP/ATP-dependent manner. HSPA8 first binds client proteins in a low-affinity, fast exchange rate ATP-state. Following binding, and with the help of accessory proteins from the HSP40 family, it hydrolyses ATP into ADP, and adopts a high affinity, slow-exchange rate ADP-state. Through the action of NEFs, it reverts to its ATP-state, thus releasing its substrate peptide [44,45].

In the case of stress, HSPA8, which is very abundant in the cell cytoplasm, shuttles between the cytoplasm and the nucleus/nucleoli, enabling HSPA8 to import client (cytoplasmic) proteins into the nucleus. This ATP-dependent mechanism is finely regulated to ensure cell protection. When the situation returns to normal, HSPA8 is released from its nuclear/nucleolar anchors and redistributes into the cytoplasm [46]. It has been recently described that a phosphopeptide called P140, known to interact with residues encompassed by the HSPA8 NBD [47,48], slows down the HSPA8 entry into the nucleus [38], but even more impressively, neutralizes the egress of HSPA8 from the nucleus to the cytoplasm in the cell recovery phase taking place after heat shock stress. This lack of the relocation of HSPA8 into the cytoplasm of heat-shocked cells alters the ability of these cells to survive when a second mild oxidative stress mimicking inflammatory conditions is applied.

By cumulating these and many other structure-activity data, we are seeing that the existence of distinct binding sites able to accommodate cooperating and competing cofactors and ligands confers upon HSPA8 a large set of functions via a spectrum of conformation which it can adopt. These sites are distributed all along the protein sequence, and the short, flexible hinge region that links the NBD and SBD is essential in this structural adaptation. As many other proteins that are composed of structural modules, HSPA8 can adopt a structure that is complementary to the surface of its interactants (ligands, cofactors, substrates), and in a dynamic and adaptive process, HSPA8 makes extensive contacts with them in a variety of functional complexes.

## 4. HSPA8 and Immune Disorders

HSPA8, the most abundant cytosolic family member, is a vital protein. HSPA8 knock-out mice cannot be created due to its key cellular functions [49], and RNA interference-based knock-down of HSPA8 results in massive cell death in various cell subtypes [50]. As such, any defect of its expression and/or localization and trafficking can lead to potentially severe pathologies. 

It is out of the scope of this review to give details on these pathophysiological situations. They are described in recent essays and compilations related to its involvement in infections, neurodegenerative diseases, cardiac diseases, stroke, metabolic diseases, cancer, asthma, aging and others [3,14,51,52,53]. Below we will focus our attention on immune disorders in which HSPA8 defaults have been described.

With the help of cytoplasmic chaperones and cofactors, HSPA8 orientates proteins towards the proteasome or to lysosomes for degradation. HSPA8 is decisive in CMA, where it represents a limiting factor for protein translocation, and also participates in the processing of targeted protein via its lysosomal form (lys-HSPA8; Figure 1). Therefore HSPA8 is central at different key steps in the presentation of peptide antigens to CD4^+^ T cells, with a potential to regulate T and B cell activation and the final secretion of antibodies by plasma cells [6,7,25,26,28,54,55,56]. HSPA8 may also have a role in antigen presentation via MHCI molecules and CD8^+^ cytotoxic T lymphocytes. Nowadays, however, the underlying mechanisms triggering these pathways remain poorly defined. Much more work is needed to understand how the selection and the processing of proteins take place, and what is the real molecular influence of cytoplasmic and lysosomal HSPA8s in these processes. It is not known, either, to what extent the quality or quantity of protein fragments generated via other autophagy pathways, such as by eMI, can be altered as a function of HSPA8 level of expression, and can possibly affect immune response.

HSPA8 expression has been seen to be altered in a number of immune disorders. Flow cytometry studies show, for example, that in the spleen of MRL/lpr lupus-prone mice, the expression of HSPA8 is raised at the surface of the B cells, T cells and specially-activated T cells, and the CD11b^+^Gr-1^+^ granulocytes/macrophages [57,58]. This overexpression of HSPA8 was seen to correlate with increased mRNA expression in MRL/lpr splenocytes. At this stage, however, it is not known whether these alterations affect all or a subset only of MRL/lpr immune cells of the spleen.

If in such diseases, it would be advisable to design some strategies of intervention aimed at decreasing the abnormally-raised expression of HSPA8, in other settings, however, an activation of HSPA8 could present a therapeutic advantage. This is the case, for instance, of the familial cold auto-inflammatory syndrome (also known as familial cold urticarial), a rare inherited disease that causes episodes of fever, skin rash and joint pain after exposure to cold temperatures. The disease is caused by a gain-of-function mutation, H443P, in the inflammasome inducer nucleotide-binding oligomerization domain-like receptors-family CARD-containing protein 4 (NLRC4). This protein is a member of the large oligomerization domain (NOD)-like receptor-family of cytoplasmic immune receptors, which upon the detection of certain pathogens or of an internal distress signal, initiates a caspase-1-mediated inflammatory response. HSPA8 interacts with both NLRC4 and mutated NLRC4. However, the H443P mutation favors the formation of a more stable complex with HSPA8 [59]. Exposure to subnormal temperature (e.g., 4 °C) decreases the interaction of mutated NLRC4 with HSPA8, leading to activation of caspase-1 and a consequent secretion of pro-inflammatory cytokines (IL-1β and IL-18). Rescuing this interaction by HSPA8 activation might represent a valuable therapeutic strategy [59].

The supply of exogenous HSPA8 or its pharmacological induction into the central nervous system could also produce favorable effects in the case of multiple sclerosis (MS). In this chronic autoimmune disease, the immune system attacks the protective sheath (myelin) that covers nerve fibers, leading to demyelination and neurodegeneration. Under normal conditions, HSPA8 acts as a chaperone for myelin basic protein, one of the two major proteins of the myelin sheath. The HSPA8 content has been found to be 30–50% lower in MS lesions, compared to normal brain tissue, with chronic lesions showing the weakest expression. This lower expression might play an important role in the permanent myelin loss in the lesions (reviewed in [60,61]). Targeting HSPA8 with chemical activators may also produce some clinical benefit in the case of amyotrophic lateral sclerosis [62].

## 5. HSPA8 Chemical Activators

**Figure 3:**
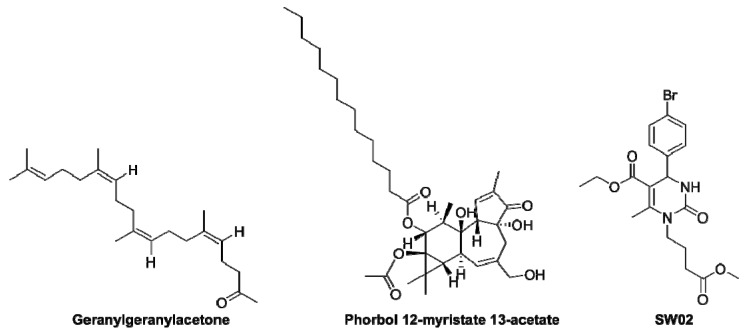
Structures of HSPA8 activators.

From the few examples given above, it emerges that HSPA8 may represent a valuable pharmacological target in a number of autoimmune situations. Surprisingly, however, when we analyze the state of the art, relatively few HSPA8 activators were explored compared to inhibitors. They are listed in Table 2 [63,64,65,66,67,68] and are shown in Figure 3. The effects of these molecules were investigated in a limited number of pathophysiological situations, and in-depth analyses of their mode of action are still lacking, especially in the context of autoimmunity. In general it is not known either, whether or not they interfere with HSPA8 by interacting directly with this chaperone.

## 6. HSPA8/HSPA8 Chemical Inhibitors

Compared to HSPA8 activators, the pipeline of HSPA8 inhibitors is much more diverse, and presents a wider range of applications. The overexpression of HSPA8 is associated with various disease phenotypes, and downregulating its activity stands as a rational way to influence the course of chronic and acute diseases, as for instance, in cancer, viral infections and neurodegenerative diseases [69,70,71]. A wide variety of molecules from natural or synthetic or semi-synthetic sources have been described (Table 3; [72,73,74,75,76,77,78,79,80,81,82,83,84,85,86,87,88,89,90,91,92,93,94,95,96,97,98,99,100,101,102,103,104,105,106,107,108,109,110,111,112]). A number of these pharmacological compounds alter the level of HSPA8 mRNA and/or protein, and do not directly bind to HSPA8. In contrast, some molecules directly interact with HSPA8 (Table 3). 

**Figure 4:**
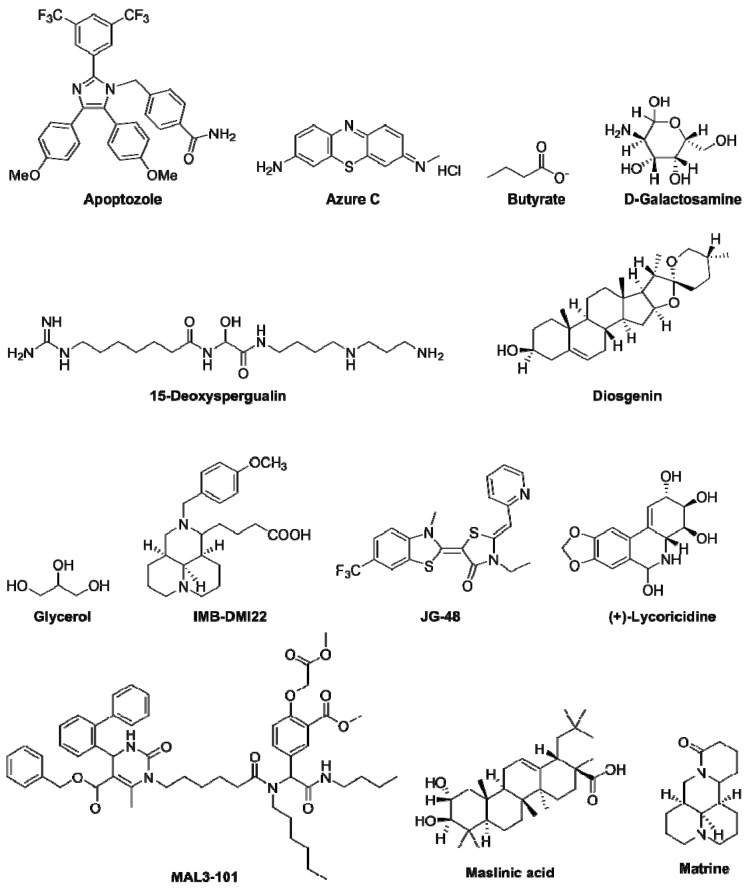

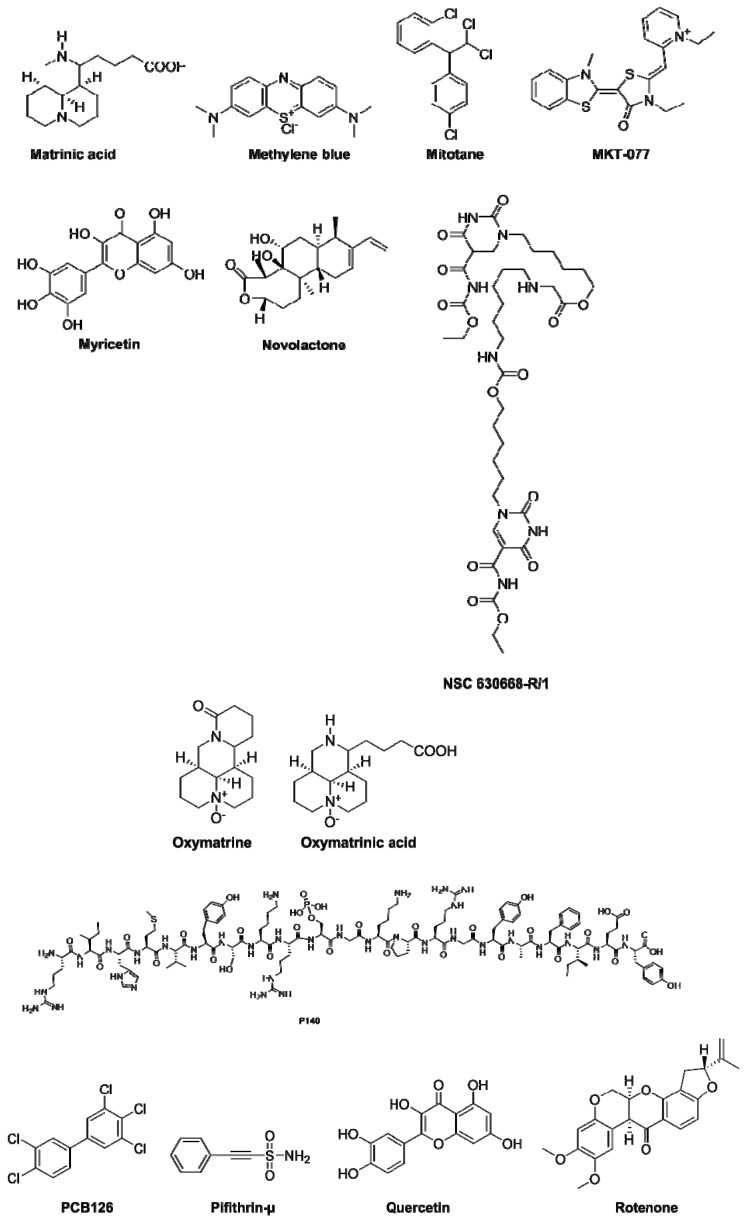

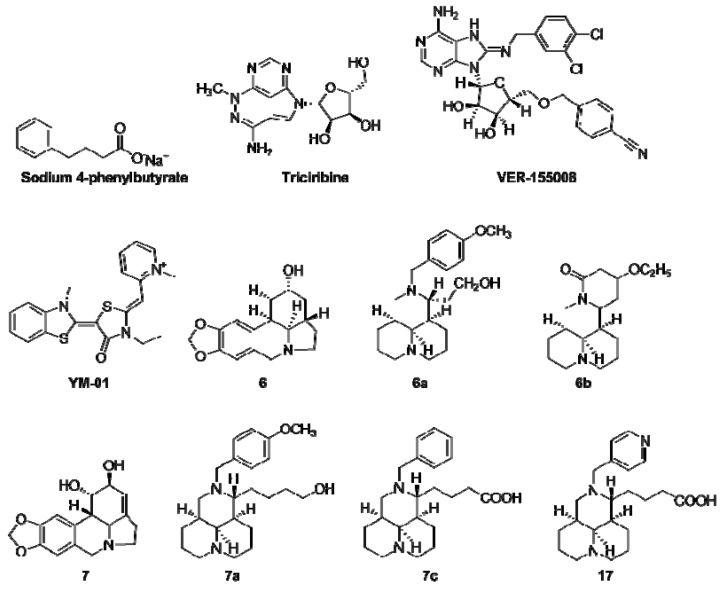
Structures of HSPA8 inhibitors. Compounds: **6a**, 12*N*-(4-Methoxybenzyl)matrinic ethanol dihydrochloride; **7a**, 12*N*-(4-Methoxybenzyl)matrinol dihydrochloride; **6**, Dehydro-1-deoxylycorine; **7**, Lycorine; **17**, 12-(4-Pyridylmethyl)matrinic acid dihydrochloride.

Most of them target the NBD domain of HSPA8, and due to similar conserved regions present in HSP70AA1 and HSPA8 [3], a number of these compounds bind both molecules, and some of them also interact with HSP90AA1 and other HSPs. In general, the fine mechanism of action underlying the inhibition of HSPA8 by these small molecules and peptides is not fully elucidated, and structure–activity relationship studies are still awaited to understand how they alter HSPA8 activity. A small number of HSPA8 inhibitors, such as 15-deoxyspergualin (15-DSG), apoptozole, peptide P140, D-galactosamine, or MAL2-11NB, an intermediate in the synthesis of MAL3-101 (Table 3; Figure 4), have been shown to display some influence on immune regulating circuits, with beneficial effects. Two of these molecules, 15-DSG and P140, have been evaluated in patients (see below). 

## 7. HSPA8/HSPA8 as a Therapeutic Target in Clinical Trials

Although HSPA8 appears as a key cellular element, especially in the endo-lysosomal pathway, and a potential central target for therapeutic applications, very few clinical trials based on HSPA8 inhibitors or activators have been performed.

Some clinical investigations were done with 15-DSG (1-amino-19-guanidino-11-hydroxy-4,9,12-triazanona-decane-10,1–3-dione; Table 3; Figure 4). DSG is a synthetic analog of spergualin, a natural product of the bacterium *Bacillus laterosporus* [113]. A long list of more stable analogs have been designed, synthesized and evaluated over years. 15-DSG is a potent immunosuppressant, which shows an immunosuppressive activity both in vitro and in vivo, affecting B lymphocytes, T lymphocytes and macrophage/monocyte functions. DSG binds to HSPA8 and HSP90, and modulates the functions of both HSPs [114]. 15-DSG blocks the NF-κB pathway and antigen presentation, causing an alteration in the activation of immune cells, notably monocytes and DCs. It inhibits AKT kinase (protein kinase B) activation and phosphatidylcholine synthesis [115]. DSG was shown to be effective in both prophylaxis and disease intervention across numerous animal models (lupus, rheumatoid arthritis, thyroiditis, graft-versus-host disease, myasthenia gravis, chronic encephalomyelitis, diabetes, vasculitis and others). Especially, it was able to suppress the progression of polyclonal B cell activation and lupus nephropathy in lupus-prone MRL/lpr mice. In a short trial including patients with systemic lupus erythematosus (SLE), two of three DSG/Gusperimus-treated lupus patients showed infectious episodes, and the assay was interrupted [116]. 

A phase-I/II study including 21 SLE patients showed better promise in terms of tolerability (although severe infections occurred in 7 of these patients) and clinical status [117]. Patients were given DSG by the subcutaneous route (starting dose at 0.5 mg/kg). Half of these SLE patients showed a complete or partial response, with a significant reduction of their proteinuria and of their daily corticosteroid (prednisolone) intake (standard of care, SOC). Gusperimus has also been evaluated in patients with glomerulonephritis (GN). In a multicenter, open-phase trial including 44 patients with various types of crescentic GN, the proteinuria of patients was significantly reduced, and their renal function, as well as their hematuria, was improved. Patients received daily doses of 0.1 or 0.2 mg/kg (the latter dose was efficient) for four weeks along with the continuation of corticosteroid therapy. These improvements persisted during the 2-month follow-up period after the discontinuation of therapy [118]. 15-DSG was also used clinically in the therapy of renal transplant rejection and Wegener’s granulomatosis [114,119,120]. Altogether, there is a body of evidence pointing to the efficacy of DSG in various pathophysiological indications including infection, disease of the immune system and inflammation. Future investigations are however eagerly expected to ensure the absence of risks to users, and to solve the existing safety/toxicity concerns.

Another therapeutic strategy that targets HSPA8 has been successfully evaluated in animal models of autoimmunity and in patients with SLE. This strategy is based upon a 21-mer peptide called P140 (because it encompasses a phosphoserine residue at position 140; Table 3; Figure 4). This peptide was initially spotted in a cellular screening assay using overlapping (non-phosphorylated) peptides covering the whole spliceosomal U1-70K protein and CD4^+^ T cells from lupus mice [121]. P140 exhibits highly favorable intrinsic physicochemical properties, it is not immunogenic [122], it is safe, and it displays no immunosuppressive activity in mouse and human settings [123]. It acts as an immunomodulator that interferes with excessive immune responses occurring in lupus and other autoimmune diseases, and considerably slows the pathophysiological process down (reviewed in [124,125,126,127]) It was shown to directly interact with HSPA8 and to inhibit the chaperone activity of the latter [47,57]. Although its location is not precisely known at the molecular level [38], the P140 binding site on HSPA8 NBD is different from the one recognized by the HSPA8 inhibitor VER-155008 [122]. As mentioned above, P140 affects the nuclear/nucleolar translocation of HSPA8 [38]. It was shown both in vitro and in vivo that P140 enters MRL/lpr spleen B cells via a clathrin-dependent pathway, and accumulates into lysosomes [48]. In vitro experiments in the NIH3T3 cell line showed a direct effect of P140 on the CMA pathway [48,58]. This effect on CMA was also supported by demonstrating that upon treatment of MRL/lpr mice with P140, the levels of the two key adaptors for CMA, LAMP-2A and HSPA8, which are overexpressed in MRL/lpr immune cells, are significantly decreased in the B cells of treated mice. Other lysosomal proteins, cathepsin L and LAMP-1, follow the same trend. P140 does not home into mitochondria, and displays no detectable effect on mitophagy [128]. P140 displays no direct effect on B cell receptor signaling in memory, naive, mature, transitional or B1 human cells, suggesting that it does not alter B cell survival and maturation in these B cell subsets [128]. However, likely as a matter of consequence resulting from its interaction with the HSPA8 chaperone, it strongly reduces the overexpression of MHCII molecules on lupus B cells acting as antigen-presenting cells, and hampers peptide–MHCII molecule loading in late lysosomal vesicles (MIIC compartment). This impressive effect shown in both mice and human settings [48,123,129,130] decelerates the complex signaling cascade leading to the final production of pathogenic autoantibodies secreted by secreting plasma cells.

In a multicenter, randomized, placebo-controlled phase-IIb study for lupus, P140/Lupuzor was found to be safe, and met its primary efficacy endpoints [131], confirming the results of a first open phase-IIa clinical trial [132] and earlier pre-clinical data generated in MRL/lpr mice. Lupuzor has been evaluated in phase-III clinical trials in the US, Europe, and Mauritius. Patients were given Lupuzor once a month by the subcutaneous route at a dose at 200 µg peptide/patient for 12 months.

Across the whole study population, in those patients who had double-stranded (ds) DNA marker autoantibodies, Lupuzor demonstrated a superior response rate over the placebo (61.5% vs. 47.3%, *p* = 0.0967), although these results were not statistically significant. Further data analysis demonstrated that in the Europe cohort (130 patients in total), Lupuzor plus SOC showed statistically significant reductions (71.1% vs. 48.8%, *p* = 0.0218) in disease activity, compared to the placebo plus SOC in 79 patients who were anti-dsDNA autoantibody positive. An open label 6-month extension study from this original phase-III trial has been performed in 2019. The results confirm the robust safety profile and tolerability of Lupuzor. Other clinical trials are being planned. Of note, P140 has been assayed in animal models mimicking other autoimmune and inflammatory conditions. Specially, it was found to be efficient in murine models of neuropsychiatric lupus [133,134], and chronic inflammatory demyelinating polyradiculoneuropathy [135]. P140 also showed remarkable effects on the salivary glands of model mice that develop a secondary Sjögren’s syndrome that is especially characterized in patients by symptoms of extensive dry eyes (xerophthalmia) and dry mouth (xerostomia) [129]. At this stage, clinical trials including affected patients will be critical to analyze the robustness of this approach in non-lupus settings.

It is expected that this review will motivate developers to pursue this line of potential therapeutic option. While there are no clinical trials currently planned or ongoing to study compounds such as geranylgeranylacetone (an HSPA1A inducer initially used to treat gastric ulcers; Table 2) or 17-(2-dimethylaminoethyl)amino-17-demethoxygeldanamycin, an HSP90-antagonist that indirectly acts on HSPA8, in stroke or related acute neurological conditions, there is hope for translation, as some of these compounds can pass the brain barrier after parenteral administration [136,137].

## 8. Conclusion and Future Prospects

Surprisingly still very few programs are centered on HSPA8 for the development of targeted therapies. However, it is becoming self-evident that this unique HSP should be central in future therapeutic strategies [1,2,3]. Although a clearer picture of the HSPA8 structure has to be better defined, especially when the protein is associated to its ligands and co-chaperones, and that some of its key functions, notably with regard to its important involvement to maintain proteostasis in the cell should be further explored, this protein plays decisive properties that deserve much attention. In autophagy processes, in particular, it acts as a real molecular rheostat that adjusts CMA substrates and therefore interferes, at an upstream level, with the full cascade of successive events that ultimately ends up with the activation or silencing of immune cell subsets, to the production of antibodies and the secretion of cytokines and chemokines that regulate the quality and extent of immune response. Yet, much has to be deciphered to understand clearly how HSPA8 recognizes and interacts with the KFERQ motif present in selected proteins intended to be processed via CMA.

In humans, the basal expression of HSPA8 is particularly high in certain organs, such as the spleen, kidney, brain and adrenal glands (as evaluated by expression levels of HSPA8 genes). This should help in targeting some organs or tissues rather than others. In contrast to other HSPs, HSPA8, which is constitutively expressed, is also particularly abundant in the cytoplasm. We have seen above that it is crucial in the protection of the cytosolic material it brings into the nucleus in case of stress [38,46]. Its expression level is increased in case of inflammation as it is the case in lupus B cells [47,57].

Recent data have demonstrated that ULK1 interacts with HSPA8, and that the KFERQ-like motif 227-QDLRL-231 in ULK1 is responsible for this binding [138,139]. This interaction is enhanced when ULK1 is phosphorylated, a modification that occurs via the mammalian target of rapamycin complex 1 (mTORC1) or by AMP-activated protein kinase. ULK1 is a serine/threonine protein kinase that plays a critical role during the initial stages of macroautophagy through the ULK1/Fip200(RB1CC1)/ATG13 complex [140]. It also regulates autophagy activity via a phosphorylation of the BECN1/VPS34/VPS15 complex [141]. 

It has been shown that when ULK1 was knocked out, the amount of LAMP2A and HSPA8 increased, strongly suggesting that CMA is activated [139]. This might constitute a basic line of possible therapeutic intervention when a reduction of CMA activity is desired.

Extracellular HSPA8 plays important metabolic roles [1]. Its level in the serum can vary in certain pathological settings [142,143]. Studies have validated the ability of extracellular HSPA8 to regulate cancer cell proliferation and sperm storage. Extracellular HSPA8 has also been seen to induce pro-inflammatory cytokines TNF-α, IL-1β and IL-6 via an activation of TLR4 in macrophages and the myocardium [144]. Regulating the expression or effects of HSPA8 in such indications might be an important therapeutic option. It may alter the binding of excessive HSPA8 with the many receptors it recognizes at the cell surface (e.g., the α-2 macroglobulin receptor CD91, CD40, some TLRs, or the lectin-like oxidized low-density lipoprotein receptor-1).

We have mentioned above that the majority of molecules that inhibit HSPA1A also target HSPA8, due to similar, conserved regions in their respective primary sequence [3]. The conformational similarity of certain HSP structural domains and modules [5] also favors these interactions. Some molecules that interact with HSP90AA1 also bind HSPA8 for the same reasons. Designing molecules that will more specially interact with HSPA8, preferably into the NBD, without affecting the other HSPs of the HSP70 family or other families, is crucial to avoid altering unwanted metabolic circuits. This requires in-depth investigations based upon advanced molecular docking and structure-based virtual screening methods to be exploited, as well as the validation of lead molecules in high-throughput biochemical and cellular screening assays. To this end, multiplex, large-scale assays should be developed to ascertain the ability of newly-discovered HSPA8 binders to specifically alter the properties of HSPA8.

In light of the vital role of autophagy in immune responses and in general in all metabolic processes of the cell, the pharmacological manipulation of autophagy elements constitutes a unique option of therapeutic intervention [126,127,145,146,147,148,149,150]. In this scheme, HSPA8 represents a hot spot of possible regulation. Future studies will have to focus on dissecting much further the molecular mechanisms underlying the intracellular and extracellular functions of HSPA8, and on investigating the effects of HSPA8 inducers and inhibitors on these properties.

## Figures and Tables

**Table 1:** Differences between HSPA1A and HSPA8 [1,2,3], (where HSP represents heat shock proteins).

Characteristics	HSPA1A	HSPA8
Molar mass	70 kDa	72 kDa
Length (amino acid residues)	641	646
Variable region	C-terminal domain	C-terminal domain
C-terminal EEVD motif	Yes	Yes
Binding of co-chaperones and other HSPs	Yes	Yes
Hydrophobic linker DL/VLLLD connecting the nucleotide and substrate binding domains	Yes	Yes
Expression	Abundant in stress conditions	Constitutively expressed and relatively less strongly expressed during stress
Association with immunogenic peptides	Yes	Moderate
Trafficking of ion channels (sodium channels)	Increased	Decreased
Interactions with lipid bilayers	Yes	Yes
Liposomal aggregation	Moderate	Yes
Involvement in drug resistance of cancer cells (e.g., leukemia cells)	Yes	No
Involvement in tumor growth	Yes	Not yet explored in depth
ATP/ADP-dependent	Yes	Yes

Abbreviations: ATP/ADP, adenosine 5’-triphosphate/diphosphate.

**Table 2:** HSPA8 activators.

HSPA8 Activator	Type of Molecule and Effects	Ref.
Geranylgeranylacetone	Acyclic polyisoprenoid derived from natural plant constituents (e.g., geranylgeraniol). Induces protein kinase C. Leads to neuroprotection against cerebral infarction in rats. Increases HSPA8 expression, which protects the mucosa from ulcers in rats. Has shown protective effects against autoimmune inflammatory diseases.	[62,63,64,65]
Phorbol 12-myristate 13-acetate	A diester of natural phorbol. Increases the amount of HSPA8 in human PBMCs via the protein kinase C pathway.	[66]
SW02	Class of dihydropyrimidines molecule. Enhances the adenosine 5’-triphosphatase (ATPase) activity of HSPA8. Inhibits protein aggregation (amyloid). Inhibits protein TAU degradation.	[67,68]

**Table 3:** HSPA8 inhibitors.

Compounds	Therapeutic Field *	Observation ^#^	References
Apoptozole	• Vaccine efficacy and cancer	Binds to the NBD of HSP70AA1 and HSPA8 and inhibits their activity. Has shown vaccine adjuvant activity (increased immune response against protein antigens) with model antigens, keyhole limpet hemocyanin and ovalbumin. Has shown antitumor effects by inducing caspase-dependent apoptosis.	[72,73,74]
15-Deoxyspergualin (15-DSG)	Cancer Immunosuppression in organ transplantation and autoimmune indications	Synthetic derivative of spergualin (bacteria-derived antibiotic). Enhances the ATPase activity of HSPA8 by binding to its EEVD domain. Inhibits antigen presentation in monocytes and DCs via the NF-κB pathway. Inhibits antigen-activated CD4^+^ T cells and reduces the polarization of IFN-γ secreting Th1 effector cells. Was shown to prevent the rejection in organ transplantation and autoimmunity.	[2,75,76,77,78,79]
D-Galactosamine	• Septic shock	Hexosamine derived from galactose Decreases HSPA8 level in the liver when co-administered with lipopolysaccharide or TNF-α (not observed when used alone).	[80]
Diosgenin	• Neurological diseases	Natural steroidal sapogenin. Inhibits HSPA8 expression in neurons (in vitro and in vivo), which is upregulated by excessive amyloid-β conditions. Binds to cell surface receptor, 1,25D3-membrane-associated rapid response steroid-binding receptor, and activates axonal regrowth.	[71]
13-Ethoxymatrine (**6b**)	• Infectious diseases	Derived from the oxymatrine. Downregulates HSPA8 mRNA levels in hepatic cells.	[81]
Glycerol	• In silico studies	A triol with a structure of propane substituted at positions 1, 2 and 3 by hydroxy groups. Inhibits HSPA8 activity. Confirmed by NMR, X-ray crystallography, and in silico docking studies.	[41]
IMB-DM122	• Infectious diseases	Semi synthetic compound derived from the matrine backbone moiety. Reduces both the mRNA and protein levels of HSPA8 in hepatocytes. Non-toxic to hepatocytes in vitro (up to 1000 µg/mL) and in vivo (up to 1000 µg/mL). 1.3-fold more potent inhibitor of HSPA8 when compared to oxymatrine.	[82,83]
JG-48	• Stability on model proteins	Synthetic derivative of MKT-077. Binds to the NBD domain of HSPA8, but not to the ATP binding subdomain region. Inhibits ATPase activity, substrate refolding and client release.	[84]
(+)-Lycoricidine	• Infectious diseases	Natural alkaloid Inhibits virus loading in host cells by reducing the HSPA8 levels.	[85,86]
MAL3-101	• Cancer	A second-generation derivative of the dihydropyrimidines Selectively binds to the NBD of HSPA8. Widely used as a chemical probe in many cancers (small-cell lung carcinoma, multiple myeloma, Merkel cell carcinoma, and others).	[87,88]
Maslinic acid	• Cancer	Natural pentacyclic triterpene. Inhibits human pancreatic cancer cells by reducing the HSPA8 expression.	[89]
Matrine	Infectious diseases Skin diseases Cancer	An alkaloid that is one of the major components in the root of the Sophora plant. Has been studied for a possible antiviral efficacy against hepatitis B and C, as well as its impact against some skin diseases and forms of cancer. Few other molecules (e.g., **6a**, **7a**, **17** in Figure 4) derived from the matrine backbone, have shown the highest anti-HCV activity (not explored for the HSPA8 activity).	[82,90,91,92,93,94]
12-N-4-methylbenzyl matrinic acid	• Infectious diseases	Matrinic acid derivative (molecule **7c** in Figure 4) Downregulates HSPA8. 2-fold potent inhibitor of HCV in hepatocytes when compared to matrinic acid.	[95]
Mitotane (Lysodren)	• Cancer	A synthetic derivative of the insecticide dichlorodiphenyl trichloroethane. Downregulates HSPA8 expression. Possesses anti-cancer activity on adrenocortical carcinoma by affecting steroidal hormonal synthesis.	[96]
MKT-077	• Cancer	Formerly known as FJ-776; a synthesized, highly water-soluble (>200 mg/mL) rhodacyanine dye. Allosteric inhibitor (ADP-bound state) of HSPA8 with potent anti-cancer properties.	[97]
NSC 630668-R/1	• Cancer	Dihydropyrimidine. Selectively binds to HSPA8 in its oligomeric state. Inhibits ATPase activity and protein translocation. Identified from an in vitro screen of molecules with an anti-tumor activity.	[98]
Oxymatrine	• Infectious diseases	As matrine, it is a natural alkaloid component extracted from the herb *Radix Sophorae flavescentes*. Reduces HSPA8 expression. Anti-HBV activity.	[83]
P140 (Lupuzor)	• Autoimmunity	Synthetic peptide corresponding to the sequence 131-151 of the U1-70K snRNP protein. Contains a phosphoserine at position 140. Binds to the NBD of HSPA8. Decreases autoantigen presentation in autoimmune diseases. Inhibits overexpression of LAMP2A in lupus B cells. Inhibits the nuclear translocation of HSPA8.	[38,47]
3,3′,4,4′,5-Pentachlorinated biphenyl 126 (PCB126)	• Zebrafish model	Derivate of polychlorinated biphenyls (dangerous environmental contaminants). Downregulates HSPA8 in the majority of vertebrates.	[99,100]
2-phenylethynesulfonamide or PES (pifithrin-μ)	• Cancer	2-phenylethyenesulfonamide Binds to the SBD of HSPs Inhibits the co-chaperones interaction with HSPs. Alters the autophagy-lysosome and proteasome degradative pathways, especially in tumors.	[101,102,103,104,105]
Quercetin	• Neurological diseases	Natural flavonoid. Decreases the synthesis of MBP in immature oligodendrocytes by inhibiting HSPA8 (no effect on other cell types, e.g., leukemia cells).	[106,107]
Rotenone	• Neurodegenerative diseases	Belongs to the retinoid family. Is naturally present in leguminosa plants; is considered to be cytotoxic. Is commonly used as an insecticide and a pesticide. Downregulates HSPA8 in neuronal cells. Has been used as a tool to induce Parkinson’s disease.	[108]
Sodium 4-phenylbutyrate	• Cystic fibrosis model	A salt of an aromatic fatty acid, 4-phenylbutyric acid. Downregulates HSPA8 in cystic fibrosis epithelial cells. Improves the degradation of the cystic fibrosis transmembrane conductance regulator mutant (∆F508-CFTR), which is altered by the association of HSPA8.	[109]
VER155008	• Cancer and neurodegenerative diseases	5′-O-[(4-Cyanophenyl)methyl]-8-[[(3,4-dichlorophenyl)methyl]amino]-adenosine. Competes with the ATP site of HSP70/HSPA8 and inhibits their function. Displays some role in apoptotic, cytostatic and cytotoxic effects on cancer cells, and also amelioration in Alzheimer’s disease.	[43,103,110,111]
YM-01	• Cancer	Chemically derived from MKT-077. Like MKT-077, it allosterically inhibits HSPA8. Has shown an anti-proliferative activity on cancer cells with non-toxic to normal cells. Many similar compounds derived from the MKT-077 are listed in [112].	[112]

Abbreviations: DCs, dendritic cells; HBV, hepatitis B virus; HCV, hepatitis C virus; INF, interferon; MBP, myelin basic protein; NF-κB, nuclear factor-kappa B; NMR, nuclear magnetic resonance; snRNP, small nuclear ribonucleoprotein; Th1, one of the two helper T cell classes; TNF, tumor necrosis factor. * The molecules tabulated in this table are all HSPA8 inhibitors. However, most of these molecules (except 15-DSG and P140) have not been evaluated in full details, and were studied in preliminary tests only. Their efficacy in vivo, especially in autoimmune diseases models, is generally unknown. ^#^ The molecular mechanism by which these small molecules affect the HSPA8 activity is available/known for a limited number of molecules only. When known, their direct binding to HSPA8 sub-regions is indicated (9/25 molecules that are listed in this table). The effect on HSPA8 of most of these investigational molecules has generally been demonstrated within in silico studies (molecular docking) or experimentally, by their ability to decrease the level of HSPA8 protein or messenger RNA expression, to affect virus replication, and to alter cancer cell viability.
